# My Physical Appearance at the Center of Others’ Concerns: What are the Consequences for Women’s Metadehumanization and Emotions?

**DOI:** 10.5334/pb.558

**Published:** 2021-03-23

**Authors:** Tina Chevallereau, Florence Stinglhamber, Pierre Maurage, Stéphanie Demoulin

**Affiliations:** 1 Louvain Social Psychology Lab, Psychological Sciences Research Institute, Université catholique de Louvain, Place C. Mercier 10, B-1348 Louvain-la-Neuve, Belgium; 2 Work, Organizational and Career Psychology Lab (Worc Psy Lab), Psychological Sciences Research Institute, Université catholique de Louvain, Place C. Mercier 10, B-1348 Louvain-la-Neuve, Belgium; 3 Louvain Experimental Psychopathology Research Group (LEP), Psychological Sciences Research Institute, Université catholique de Louvain, Place C. Mercier 10, B-1348 Louvain-la-Neuve, Belgium

**Keywords:** sexual objectification, dehumanization, metadehumanization, emotions

## Abstract

Despite the frequency of women’s exposure to sexually objectifying behaviors in their daily life (e.g., through comments on their appearance, gazing or touching), no previous work has investigated how such a focus on their physical appearance influences women’s meta-perceptions. Capitalizing on recent studies showing that sexually objectified women are dehumanized by both male and female participants, the present paper investigates women’s metadehumanization (i.e., their perceptions of being viewed as less than fully human) and its emotional consequences following interpersonal sexual objectification. In three studies, we showed that when an interaction partner focuses on their physical appearance, women report higher levels of metadehumanization, as well as increased anger and sadness, than when the partner focuses on non-physical parts. Theoretical and empirical contributions of the present findings are discussed.

## Introduction

The importance of physical appearance in Western societies has led scholars to investigate its antecedents and consequences. In this context, the focus on physical appearance has mainly been referred as (sexual) objectification (Fredrickson & Roberts, 1997), although objectification can take multiple forms. Indeed, researchers propose that objectification occurs when an individual is perceived from the view point of their utility to achieve people’s needs and goals (Gruenfeld, Inesi, Magee, & Galinsky, 2008; Nussbaum, 1995). Given such definition, the consequences of objectification have mainly been studied in workplace, slavery, or sexual contexts. For instance, workplace objectification occurs when workers are used as means to production while sexual objectification appears when lovers respectively emphasize on and benefit from each other’s bodies. The latter case would particularly affect women, placing their physical appearance at the center of others’ concerns (Fredrickson & Roberts, 1997). Fredrickson and Roberts (1997) proposed that sexual objectification occurs in both mainstream media and interpersonal interactions, through a focus on women’s physical appearance. Although underdeveloped, studies that investigated interpersonal sexual objectification have shown, that such focus on physical appearance lead women to experience body dissatisfaction or body shame (e.g., Calogero, 2004; Moya-Garófano, Rodríguez-Bailón, Moya, & Megías, 2018), self-objectification (e.g., Fairchild & Rudman, 2008; Garcia, Earnshaw, & Quinn, 2016; Holland, Koval, Stratemeyer, Thomson, & Haslam, 2017), and reduced cognitive performance (e.g., Garcia et al., 2016; Gay & Castano, 2010; Gervais, Vescio, & Allen, 2011; Kahalon, Shnabel, & Becker, 2018). However, little is known regarding the impact of such a focus on women’s meta-perceptions and emotions.

The present paper fills this gap by investigating the effect of a focus on physical appearance in interpersonal encounters on metadehumanization (i.e., the perceptions of being viewed as less than fully human; Kteily, Hodson, & Bruneau, 2016) and finally negative emotions. Despite the results of studies showing that sexually objectified female targets are denied human characteristics (Heflick & Goldenberg, 2009; Morris, Goldenberg, & Boyd, 2018; Vaes, Paladino, & Puvia, 2011), no previous research investigated the victims’ perspective following sexual objectification experiences. Hence, we hypothesized that when women face a partner focusing on their physical appearance, they experience being dehumanized and thus report higher metadehumanization levels.

In addition, based on Bastian and Haslam (2011) and Zhang, Chan, Xia, Tian, and Zhu (2017)’s studies which showed that metadehumanization increases negative emotions among victims and particularly feelings of anger, sadness and guilt, we hypothesized that a focus on physical appearance will induce negative emotional responses via metadehumanization. Consequently, the mediating role of metadehumanization in the relationship between interpersonal sexual objectification and negative emotions will be investigated.

## Sexual Objectification as a Focus on Physical Appearance

Physical appearance is a key determinant of social, economic, and work-related outcomes (e.g., Cash, Gillen, & Burns, 1977; Dion, Berscheid, & Walster, 1972) and its effects on one’s life are more pronounced for women (Feingold, 1990, 1991; Hernández-Julián & Peters, 2017; Jackson, Sullivan, & Hymes, 1987; Sprecher, 1989). In 1997, Fredrickson and Roberts developed Objectification Theory, which aimed at examining the antecedents and consequences of being treated as a sexual object in a society that values women’s physical appearance. These authors define sexual objectification as “*the experience of being treated as a body (or collection of body parts) valued predominantly for its use to (or consumption by) others*” (Fredrickson & Roberts, 1997, p.174). Such a focus on physical appearance is spread both through media (i.e., in which women are presented as sexual objects, dressed provocatively or in suggestive positions) and social interactions (i.e., in which women are gazed at, visually inspected or in which they receive comments on their physical appearance).

Following Fredrickson and Roberts’ seminal work, many scholars have started to investigate the consequences of sexual objectification for women. While many studies concentrated on the effects of the exposure to sexually objectifying media, fewer research examined sexual objectification in interpersonal encounters (Gervais, Sáez, Riemer, & Klein, 2020). In 2007, Kozee, Tylka, Augustus-Horvath and Denchik developed The Interpersonal Sexual Objectification Scale (ISOS). This scale makes the distinction between body evaluation behaviors (e.g., gazing, catcalls, sexual remarks and comments) and unwanted sexual advances (e.g., unwanted touching, sexual harassment). Research has shown that women frequently experience both forms of sexually objectifying behaviors (Fairchild & Rudman, 2008; Holland et al., 2017; Macmillan, Nierobisz, & Welsh, 2000; Swim, Hyers, Cohen, & Ferguson, 2001). For instance, women report being confronted with sexual objectifying comments 1 or 2 times a week (Swim et al., 2001). Holland et al. (2017) investigated a broader range of body evaluation behaviors (i.e., gazing, sexual remarks, catcalls/wolf whistles) and found that women report being the target of objectification behaviors once every two days. Studies on sexual harassment (Fairchild, 2010) and unwanted touching (Hayden, Graham, & Lamaro, 2016) also put forward that women are frequently exposed to such behaviors (see also, Gelfand, Fitzgerald, & Drasgow, 1995).

Research on the consequences of interpersonal sexual objectification has manipulated the focus on physical appearance in a variety of ways (e.g., appearance-related comments or questions, anticipated or experienced male gaze; Calogero, 2004; Calogero et al., 2009; Gervais et al., 2011; Lameiras-Fernández et al., 2018; Moya-Garófano et al., 2018). Several research findings showed that the effects of focus on physical appearance depend on women’s (emotional) reactions (Moya-Garófano et al., 2018) and the importance they allocate to their physical appearance, that is, Trait Self-Objectification levels (i.e., TSO, here after) (e.g., Calogero, Herbozo, & Thompson, 2009; Gay & Castano, 2010; Kahalon et al., 2018).

Indeed, while Herbozo and Thompson (2006) highlighted the positive effects of appearance-based compliments on body satisfaction, self-esteem, and eating disorders, other studies qualified this result. For instance, Tiggemann and Boundy (2008) showed that compliments on appearance increase body shame for women with high TSO. Calogero et al. (2009) found similar results, showing that women with high TSO report higher levels of body surveillance and body dissatisfaction, independently of the feeling (positive vs. negative) caused by appearance-related comments (Calogero et al., 2009). Considering the effects of women’s (emotional) reactions, Moya-Garófano and collaborators (2018) found that women who react with happiness, empowerment, and low levels of anger to appearance-related comments also report higher body shame and body surveillance. Fairchild and Rudman (2008) also showed the importance of women’s reactions following sexually objectifying behaviors from strangers (i.e., exposition to appearance-focus behaviors such as catcalls, whistles or stares). Particularly, stranger harassment increases self-objectification among women who use passive coping strategies (i.e., ignoring or denying the harassment) or self-blame. Finally, recent research findings highlighted the key role of psychological intimacy with the perpetrators of sexually objectifying comments, showing that positive comments from romantic partners are perceived as less objectifying and more enjoyable than those from colleagues, strangers, and friends (Lameiras-Fernández, Fiske, González Fernández, & Lopez, 2018). All in all, these research findings highlighted the negative consequences of interpersonal sexual objectification on body-related variables (see also, Calogero, 2004).

Researchers also considered the negative effect of sexually objectifying behaviors on cognitive performance. Recently, Kahalon et al. (2018) observed a vicious effect of appearance compliments on mood and cognitive performance. Indeed, if compliments on appearance increase mood among participants with high TSO (Study 1; see also, Fea & Brannon, 2006), such comments decrease participants’ cognitive performance. The negative effect of appearance focus on cognitive performance has also been put forward through exposition to male gaze, which tend to decrease cognitive performance for women with high TSO (Gay & Castano, 2010).

For instance, Woodzicka and LaFrance (2005) showed that when women are asked sexual questions during a job interview, they have a reduced performance (i.e., spoke less fluently, gave lower quality answers) compared to women who are asked surprising but non-sexualized questions (see also, Wiener, Gervais, Allen, & Marquez, 2013). These results are consistent with Saguy, Quinn, Dovidio, and Pratto (2010)’s findings, showing that women narrow their presence in social interaction (i.e., spend less time talking) when anticipating a male gaze.

Interestingly, research examining the effects of interpersonal encounters focused on physical appearance on women’s reactions to their interaction partner is inconclusive. On the one hand, Gervais et al. (2011)’s studies indicated that, when confronted with an objectifying gaze, women are still motivated to interact with their objectifying partner. On the other hand, Teng, Chen, Poon, and Zhang (2015) found that receiving sexually objectifying remarks from an interaction partner decrease the perceived likability of the partner, leading to a reduced desire of affiliation with him.

Albeit few in numbers, these research findings suggest that being the target of a focus on one’s physical appearance in social interactions has a strong and negative influence on body image and cognitive-related variables. Those negative effects of interpersonal sexual objectification on these variables appear in spite of its somewhat unexpected positive effect on mood. Indeed, some studies showed that receiving a compliment on appearance leads the participant to report less negative mood (Tiggemann & Boundy, 2008), particularly for women with high TSO (Fea & Brannon, 2006). Unfortunately, much less is known about its consequences on women’s meta-perceptions. In the following section, we suggest that these meta-perceptions are likely to take the form of metadehumanization.

## Metadehumanization and Focus on Physical Appearance

Dehumanization refers to the perception of a group or an individual as less human than oneself. According to the dual model of dehumanization (Haslam, 2006) people may be dehumanized in two different ways. The denial of human uniqueness characteristics (e.g., civility, refinement, maturity, rationality) leads to *animalistic dehumanization* whereas the denial of human nature characteristics (e.g., emotional responsiveness, interpersonal warmth, agency, cognitive openness) leads to *mechanistic dehumanization*. Although the dual model of dehumanization (Haslam, 2006) is frequently used by researchers, the two facets have rarely been found through exploratory and confirmatory factorial analyses (Bastian & Haslam, 2010; Loughnan, Baldissarri, Spaccatini, & Elder, 2017) or studied at the same time (Zhang et al., 2017). Moreover, when researchers used the two dimensions in their design and conducted factorial analyses, the two dimensions did not systematically emerge (Bastian et al., 2012a; Bastian, Jetten, & Radke, 2012b; Demoulin et al., 2020; Tang & Harris, 2015).

After decades of empirical works on the perpetrators of dehumanization (for reviews, see Haslam & Loughnan, 2014; Haslam & Stratemeyer, 2016), scholars have now started to focus on dehumanization’s victims (Demoulin, Maurage, & Stinglhamber, in press; see also Fontesse, Demoulin, Stinglhamber, & Maurage, 2019). In particular, Kteily et al. (2016) suggested to consider metadehumanization (i.e., the experience of being perceived as less than a human by others). Research on the determinants of metadehumanization has shown that people feel dehumanized when they face interpersonal maltreatments such as social ostracism, exploitation or humiliation (Bastian & Haslam, 2011), abusive supervision (Caesens, Nguyen, & Stinglhamber, 2019), or rape and sexual harassment (Moor, Ben-Meir, Golan-Shapira, & Farchi, 2013). Other scholars have further suggested that interpersonal maltreatments lead to metadehumanization because targets’ fundamental human needs are being put at stake (Demoulin et al., 2020; see also Fontesse et al., 2019). Bastian and Haslam (2010) manipulated social ostracism either through autobiographical recalls, asking participants to report situations in which they were socially excluded (Study 1) or through the Cyberball game, in which participants were excluded to an online ball-toss game (Study 2). The authors found that social ostracism (vs. inclusion or control) negatively impacts participants’ fundamental human needs. Similarly, Yang and collaborators found that powerless people, whose needs for control and autonomy are thwarted, experience dehumanization from powerful people (Yang, Jin, He, Fan, & Zhu, 2015). More generally, in clinical contexts, patients feel dehumanized when caregivers consider their needs as secondary or unimportant (Raja et al., 2015).

Metadehumanization is particularly likely to arise when women face sexually objectifying instances for two reasons. First, and most obviously, sexual objectification occurs when people are considered only through their physical appearance. In such circumstances, fundamental human needs are probably unsatisfied or even thwarted. Moreover, sexual objectification practices can be considered as instances of interpersonal maltreatments as it is the most frequent form of gender discrimination for women (Swim et al., 2001). Miles-McLean and colleagues (2015) showed that the more women are confronted with unwanted sexual advances the more they report trauma symptoms (e.g., nightmares, sexual problems).

Moreover, research has shown that people (from both genders) attribute fewer human characteristics to sexually objectified female targets compared to non-objectified ones (Bongiorno, Bain, & Haslam, 2013). In the same vein, Vaes et al. (2011) showed that sexually objectified female targets are less implicitly associated with human-related concepts compared to both non-objectified female targets and (non-)objectified male targets. Heflick and Goldenberg (2009) found that sexually objectified female targets are denied human nature characteristics, which indicates mechanistic dehumanization.

In line with the above reported literature, we hypothesize that, when experiencing a focus on their physical appearance during interpersonal encounters, women would report feeling dehumanized by their interaction partner, i.e., metadehumanization. Such metadehumanization would come with consequences because it could, among other effects, influence the emotional experiences of these women.

## Emotional Consequences of Metadehumanization

Metadehumanization has a wide range of negative consequences, including aggression tendencies (Kteily et al., 2016), victims’ well-being impairment (Caesens, Stinglhamber, Demoulin, & De Wilde, 2017), reduced self-esteem (Demoulin et al., 2020; Fontesse et al., 2019), increased behavioral inhibition (Moor et al., 2013) and reduced use of functional coping strategies (Demoulin et al., 2020). These results are consistent with the more general findings that negative meta-perceptions impair interpersonal relationships (Owuamalam, Issmer, Zagefka, Klaßen, & Wagner, 2014; Vorauer, Main, & O’Connell, 1998). For instance, people tend to reciprocate the negative meta-perceptions with negative evaluation, endorsement of hostile behaviors and anger (Kamans, Gordijn, Oldenhuis, & Otten, 2009; Owuamalam, Tarrant, Farrow, & Zagefka, 2013).

More importantly for the present research, metadehumanization affects people emotional reactions. For instance, Bastian and Haslam (2011) showed that animalistic and mechanistic forms of metadehumanization predict specific emotional consequences. While animalistic metadehumanization leads people to report shame and guilt, mechanistic metadehumanization triggers sadness and anger. When attempting to replicate these results, Zhang et al. (2017) found that mechanistic metadehumanization indeed increases sadness but not anger, whereas animalistic metadehumanization indeed increases shame but also sadness. More importantly, the effects of metadehumanization held while controlling for more general negative evaluations.

In the present research, we hypothesize an increase in metadehumanization as a result of the attention paid to their physical appearance in women’s interpersonal interactions, which leads to negative emotions, such as anger, sadness and guilt. Given the divergent findings reported in previous studies, no specific hypotheses are proposed regarding the differential modifications related to specific negative emotions.

## Overview of the Studies

In three studies, we explored the relationships between the experience of being confronted with sexually objectifying behaviors (i.e., focus on physical appearance), metadehumanization, and emotions. Study 1 tested the hypothesis that when women face an interaction partner focusing on their body, they report higher metadehumanization levels compared to a control condition. Although most studies highlighted the negative effects of interpersonal sexual objectification, other research found that appearance-related comments may elicit positive emotional outcomes (Fea & Brannon, 2006; Herbozo & Thompson, 2006) or even enjoyment (Liss, Erchull, & Ramsey, 2011; Sáez, Valor-Segura, & Expósito, 2019). Hence, in Study 1, we measured women’s appreciation of the situation they face to account for such potential valence effects.^1^ Studies 2a and 2b also investigated the link between the focus on physical appearance and metadehumanization, together with the emotional consequences of metadehumanization. Based on Bastian and Haslam (2011) and Zhang et al. (2017)’s studies, we hypothesized that metadehumanization would trigger negative emotional reactions among victims. Finally, we examined the indirect effect of a focus on one’s physical appearance on negative emotions via metadehumanization. Data of all three studies are available on OSF at *https://osf.io/mkbrc/?view_only=ab7c2a0fc1e44473ba10f6580804d5df* and the English version of the survey flow is available on OSF through the following link *https://osf.io/evzu9/?view_only=9682664eec8a44b18ab2307d5a875cc1*.^2^

## Study 1

### Method

#### Participants

We recruited 142 female participants through the French-speaking crowdsourcing platform Foule Factory. Data from two participants were discarded because they failed attentional check questions (i.e., “*It is important that you pay attention to our survey. Please tick “Strongly disagree” for this statement*”). The final sample was composed of 140 participants. Their age ranged from 19 to 68 years (*M* = 40.04 years, *SD* = 12.00). Each participant was randomly assigned to one of the two experimental conditions (70 participants per condition). Ninety-five percent of participants were French-native speakers.

#### Procedure and Materials

**Manipulation of physical appearance focus.** Participants took part in a study named “Impression formation processes in virtual meetings”. Participants were told that they would be confronted to a new dating site working in such a way that users have to select questions they would like to ask their suitors for the future discussion. Although participants had access to all potential questions that the future partner could ask, questions presumably selected by the male interaction partner differed as function of condition assignment. In the focus on physical appearance condition, Antoine, a user of the dating site, ticked 4 questions related to physical appearance (e.g., “*Do you usually use make up to be feminine?*”) and 3 to other domains (e.g., family and friends, professional sphere, hobbies, “*Do you have good relationships with your parents?*”). In contrast, in the control condition, Antoine ticked only 1 question related to physical appearance and 6 in other domains. These questions are presented to participants as the ones Antoine would like to ask them if they agree to discuss with him. Before being exposed to the manipulation, participants also read an introduction written by Antoine. As function of condition assignment, participants read: “*Hello, my name is Antoine. Generally, I attach importance to the personality of the person I am in relationship with, but also to her physical appearance (vs. interests and hobbies): if a woman does not appeal to me (vs. share similarities with me), I am afraid it would not work between us.*».

**Manipulation check.** After the manipulation, participants had to indicate their level of agreement on a 7-point Likert scale ranging from 1 “*strongly disagree*” to 7 “*strongly agree*” to 4 items (e.g., “*I think that Antoine was mainly interested in my physical appearance*”). These items served as a manipulation check.

**Questions’ appreciation.** Participants had to indicate their appreciation of Antoine’s questions through two items (e.g., “*The questions Antoine asked me were relevant*” or “*I appreciated the questions Antoine asked me*”) using a 7-point Likert scale ranging from 1 “*strongly disagree*” to 7 “*strongly agree*”.

**Metadehumanization.** We used a slightly adapted version of Demoulin et al. (2020)’s scale to assess participants’ levels of animalistic and mechanistic metadehumanization. Eight items evaluated animalistic metadehumanization (e.g., “*Antoine thinks that women are under-evolved*”) and 8 items measured mechanistic metadehumanization (e.g., “*Antoine thinks that women are superficial, is not concerned about who they are inside*”). For each item, participants indicated their level of agreement using a 7-point Likert scale ranging from 1 “*strongly disagree*” to 7 “*strongly agree*”.

### Results

#### Preliminary Analyses

A principal component analysis was conducted on the metadehumanization scale to check if the two dimensions of dehumanization emerged. The results revealed a one-factor structure explaining a total of 72.98% of variance, with factor loadings ranging from .758 to .913 on the single factor. Analyses also revealed a good internal consistency, .97. Hence, we merged all metadehumanization items (i.e. animalistic and mechanistic ones) to create a single metadehumanization index.

#### Main Analyses

**Manipulation check.** We conducted a one-way ANOVA on the focus on physical appearance manipulation check. Results indicated that our manipulation was successful, as participants reported higher levels of focus on their physical appearance in this condition (*M* = 5.36, *SD* = 1.31) than in the control one (*M* = 2.55, *SD* = 1.11), *F*(1,139) = 185.24, *p* < .001, η^2^*_p_* = .57.

**Questions’ appreciation.** Participants reported that Antoine’s questions were more relevant and appreciable in the control (*M* = 4.82, *SD* = 1.59) than in the focus on physical appearance condition (*M* = 3.30, *SD* = 1.49), *F*(1,139) = 33.59, *p* < .001, η^2^*_p_* = .19.^3^

**Metadehumanization.** As expected, a one-way ANOVA revealed a main effect of condition, *F*(1,139) = 46.04, *p* < .001, η^2^_p_ = .25, indicating higher metadehumanization in the focus on physical appearance condition (*M* = 3.25, *SD* = 1.39) than in the control one (*M* = 1.85, *SD* = 1.03). Importantly, the effect of the focus on physical appearance on metadehumanization remained significant after controlling for questions’ appreciation, *F*(1,139) = 16.70, *p* < .001, η^2^_p_ = .10. After data collection, we ran sensitivity analyses using G*Power in order to know if our sample size was large enough to detect a small to medium effect size f. The results showed that with a power set at .80, an alpha of .05 and 140 participants for two conditions, we had a probability of 80% to detect a minimal effect size f of .23.

## Discussion

The purpose of Study 1 was to investigate the metadehumanization consequence of the focus on physical appearance during social interactions. As expected, our study shows that when women face an interpersonal encounter in which a male partner focuses on their physical appearance, they feel dehumanized. In other words, being questioned on their physical appearance leads women to think that the questioner denies their human characteristics and treats them as less than full human beings. Importantly, this effect persists over and above the general negative evaluation of the experience.

Exploratory factor analyses revealed that animalistic and mechanistic metadehumanization items loaded on a single-factor solution and that the two dimensions were strongly correlated (*r* = .89**). Hence, to analyze the effects of focus on physical appearance on metadehumanization levels, we chose to create a single metadehumanization index. The latter result was expected given that past studies frequently collapsed the two dimensions into a single one due to one-factor solutions and strong correlations between them (Bastian et al., 2012a; Bastian et al., 2012b; Tang & Harris, 2015).

The encouraging results of Study 1 needed replication. Studies 2a and 2b thus used a similar design, but also explored the consequences of metadehumanization, and centrally victims’ emotional reactions to the dehumanizing situation. Based on Bastian and Haslam (2011) and Zhang et al., (2017), we hypothesized that a focus on one’s physical appearance would increase women’s negative emotions (anger, sadness, and guilt) via the metadehumanization it elicits.^4^

## Studies 2a-b

### Method

#### Participants

Power analyses determined sample sizes. Based on the effect size found in the first study when considering the effect of interpersonal focus on physical appearance on metadehumanization (η^2^_p_ = .25), G*Power recommended to recruit 128 participants to provide 80% of statistical power. Hence, for Study 2a, 130 female participants were recruited through the crowdsourcing platform Prolific Academic (82% of which are Native English speakers). Data from 10 participants were discarded because they failed to answer to attentional check questions. The final sample was composed of 120 participants, with a mean age of 36.20 years (*SD* = 12.88). Each participant was randomly assigned to either the control (*n* = 58) or the focus on physical appearance (*n* = 62) condition.

For Study 2b, we also considered recruiting 130 female participants. However, due to limited recruiting opportunities, only 111 participants were recruited through our university’s pool of student participants. Ninety-five percent of the participants were Native French speakers. Data from five participants were discarded because they failed to correctly answer to attentional check questions. The final sample was composed of 106 participants. Their mean age was 20.46 (*SD* = 1.44). Here again, participants were randomly assigned to one of two conditions: 54 participants were in the focus on physical appearance condition and 52 participants in the control one.

#### Procedure and Materials

**Manipulation of the focus on physical appearance.** The manipulation was identical to Study 1.

**Dependent variables: metadehumanization and emotions.** As in Study 1, we used the same 16 items to assess metadehumanization (Demoulin et al., 2020). Then, feelings of anger, sadness and guilt were measured with The Positive and Negative Affect Schedule – Expanded Form (PANAS-X; Watson & Clark, 1999). Participants were invited to indicate how much they felt angry (6 items: angry, hostile, irritable, scornful, disgusted, loathing), sad (4 items: sad, blue, downhearted, lonely) and guilty (6 items: guilty, ashamed, blameworthy, angry at self, disgusted with self, dissatisfied with self) during the reading of the selected questions. Participants had to indicate to what extend they felt each emotion on a 5-point Likert scale ranging from 1 “*Not at all*” to 5 “*Extremely*”.

### Results

#### Preliminary Analyses

A principal component analysis on the metadehumanization scale confirmed the factor structure found in Study 1 for both Studies 2a-b. All items loaded on one single factor, explaining 65.74% of variance for Study 2a and 59.08% of variance for Study 2b. All factor loadings ranged from .699 to .873 (Study 2a) and from .612 to .835 (Study 2b) on the unique factor solution and further analyses revealed a good internal consistency (α = .96, α = .95, for Study 2a and 2b, respectively). As a consequence, and similarly to Study 1, the metadehumanization scale was considered as a unidimensional construct and items of animalistic and mechanistic metadehumanization were collapsed to create a unique index of metadehumanization.

Analyses also revealed a good internal consistency for all three emotions, anger (α = .93, Study 2a; α = .91, Study 2b), sadness (α = .81, Study 2a; α = .72, Study 2b) and guilt (α = .90, Study 2a; α = .80, Study 2b).

#### Main Analyses

**Manipulation check.** A one-way ANOVA confirmed the efficiency of our manipulation: participants reported higher levels of focus on physical appearance in this condition (*M* = 5.76, *SD* = 1.06, *M* = 5.52, *SD* = 1.04, in Studies 2a and 2b, respectively) than in the control one (*M* = 3.34, *SD* = 1.33, *M* = 2.49, *SD* = .91, in Studies 2a and 2b, respectively), Study 2a: *F*(1,119) = 120.99, *p* < .001, η^2^_p_ = .50, Study 2b: *F*(1,105) = 251.75, *p* < .001, η^2^_p_ = .70.

**Metadehumanization.** A one-way ANOVA was conducted on metadehumanization. Analyses revealed a main effect of focus on physical appearance on metadehumanization, *F*(1,119) = 17.54, *p* < .001, η^2^_p_ = .12 (Study 2a), *F*(1,105) = 27.95, *p* < .001, η^2^_p_ = .21 (Study 2b). As in Study 1, participants reported higher levels of metadehumanization in the focus on physical appearance condition (*M* = 3.90, *SD* = 1.25; *M* = 3.02; *SD* = 1.12) than in the control condition (*M* = 2.93, *SD* = 1.25; *M* = 1.98; *SD* = .86).

**Emotions.** One-way ANOVAs tested the effect of interpersonal focus on physical appearance on negative emotions (anger, sadness, guilt). Participants reported higher levels of anger when they were confronted with a focus on physical appearance (*M* = 2.21, *SD* = 1.21, *M* = 3.13, *SD* = 1.52, in Studies 2a and 2b, respectively) than in the control condition (*M* = 1.54, *SD* = .70, *M* = 1.31, *SD* = .43, in Studies 2a and 2b, respectively), *F*(1,119) = 13.34, *p* < .001, η^2^_p_ = .10 (Study 2a), *F*(1,105) = 69.15, *p* < .001, η^2^_p_ = .39 (Study 2b). Moreover, sadness was also higher in the focus on physical appearance condition (*M* = 1.79, *SD* = .92, *M* = 2.25; *SD* = 1.21, in Studies 2a and 2b, respectively) than in the control condition (*M* = 1.50, *SD* = .73, *M* = 1.51; *SD* = .76, in Studies 2a and 2b, respectively), *F*(1,119) = 3.61, *p* = .06, η^2^_p_ = .03 (Study 2a), *F*(1,105) = 14.05, *p* < .001, η^2^_p_ = .11 (Study 2b). However, we found diverging results between Studies 2a and 2b on guilt feelings. Particularly, for Study 2a, we did not find any difference on guilt between the two conditions, *F*(1,119) = .88, *p = .34*. In contrast, in Study 2b, participants reported higher levels of guilt in the focus on physical appearance condition (*M* = 1.30, *SD* = .62) than in the control (*M* = 1.10, *SD* = .24), *F*(1,105) = 4.66, *p* = .03, η^2^_p_ = .04.

**Mediation analyses.** Using bootstrap analyses, we tested the mediating role of metadehumanization in the relationship between interpersonal focus on physical appearance and anger/sadness (Hayes, 2013; macro PROCESS, model 4; resample = 5,000 iterations). In Study 2a, the effects of focus on physical appearance on feelings of anger and sadness were both fully mediated by metadehumanization, indirect effect = .43, 95% CI = [.21; .68] & .28, 95% CI = [.13; .46], respectively. In Study 2b, metadehumanization partly mediates the effects of focus on physical appearance on anger and sadness, indirect effect = .41, 95% CI = [.13; .77], indirect effect = .21, 95% CI = [.02; .44], respectively. Finally, for Study 2b, we tested the mediating role of metadehumanization when considering the effect of focus on physical appearance on guilt. The indirect effect did not reach significance, indirect effect = .05, 95% CI = [–.03; .16], indicating that the effect of focus on physical appearance on guilt was not explained by metadehumanization.

## Discussion

Studies 2a and 2b first aimed at replicating the effect of interpersonal appearance-focused behaviors on metadehumanization. Consistent with results of Study 1, women report being considered as less than fully human when confronted with a man asking questions related to their physical appearance at the expense of other domains.

Studies 2a and 2b also investigated the negative emotional consequences of interpersonal objectification experiences, and the mediating role of metadehumanization in these relationships. Both studies highlighted the mediating role of metadehumanization in the relation between a focus on physical appearance and anger and sadness. Nevertheless, while metadehumanization totally mediate the effect of objectification on emotions in Study 2a, it only partially does so in Study 2b, where we also found that interpersonal objectification increase guilt feelings independently of metadehumanization. As a whole though, the main results robustly replicate across samples differing in age, mother-language and nationality.

## General Discussion

The goal of the present research was twofold. First, we investigated metadehumanization as a consequence for women facing a focus on their physical appearance. Second, we explored the mediating role of metadehumanization in the relationship between interpersonal focus on physical appearance and negative emotions. Our three studies corroborated our hypotheses showing that when women are confronted with a focus on their physical appearance, they report feeling dehumanized by their interlocutor. Further, our studies provided support for the mediating role of metadehumanization in the occurrence of anger and sadness feelings following experiences focusing on physical appearance. As a whole, the present studies put forward the key role metadehumanization plays between focus on physical appearance experiences and the rise of some negative emotions.

It should be noted that results on guilt were inconsistent across studies, as higher guilt level in the focus on physical appearance condition was only observed in Study 2b (and not in Study 2a). Moreover, the increase of guilt feelings in Study 2b occurred independently of metadehumanization. Past research frequently highlighted the occurrence of self-conscious emotions such as self-blame, shame and guilt following sexual objectification experiences. Particularly, extreme violent forms of sexual objectification (e.g., sexual harassment, sexual assault) increase such self-conscious emotions (Vidal & Petrak, 2007; Weiss, 2010). However, this is the first time that guilt feelings were reported following a subtle, everyday form of sexual objectification that are appearance-related questions for women. The difference observed between Studies 2a and 2b on guilt feelings cannot be addressed on the basis of the data collected here. Nevertheless, the age difference between Studies 2a (*M* = 36.20) and 2b (*M* = 20.46) is not negligible and may be informative. For instance, the familiarity with online dating is probably more common among young people and could thus impact the emotional reactions of participants. Future studies are needed to answer these questions.

Our results bring new insights for both interpersonal sexual objectification and metadehumanization. Firstly, aside from the well-known consequences that sexually objectifying practices have on women (Fredrickson & Roberts, 1997; Moradi & Huang, 2008; Roberts, Calogero, & Gervais, 2018), our studies explored for the first time its effect on metadehumanization from the women’s perspective. Meta-perceptions in general, and metadehumanization in particular, have rarely been considered in interpersonal sexual objectification contexts, which is problematic for two reasons. First, metadehumanization has important consequences for the way interactions evolve (Andrighetto, Riva, Gabbiadini, & Volpato, 2016; Kteily et al., 2016). Second, by confining women to the status of victims passively and unconsciously incorporating their role as sexual objects in their self-concept, research has somewhat failed to consider the full spectrum of sexual objectification’s consequences. Scholars have advocated for a better consideration of women’s perspective in sexual objectification. Research has, for instance, recently suggested that (some) women might be very well aware that they could use their sexuality as a way to gain power over men (Erchull & Liss, 2013), benefit from a positive sexual esteem (Liss et al., 2011), and a better sexual satisfaction (De Wilde, Casini, Wollast, & Demoulin, 2019). Future studies should thus consider women’s meta-perceptions in sexual objectification settings because they might affect the way women will react to objectifying instance. Indeed, women’s meta-perceptions following sexual objectification experiences might mediate their attitudes and reactions towards sexual objectification and its associated consequences.

Secondly, the present studies provided support for the idea that interpersonal sexual objectification increases anger and sadness via women’s perception that their interlocutor denies them full humanness (i.e., metadehumanization). This result is important as emotions elicit specific behavioral tendencies (Shepherd, 2019). In particular, anger triggers active action tendencies: When considered at the group-based level, anger produces, for instance, an increase in collective actions (Chaudoir & Quinn, 2010; Guizzo, Cadinu, Galdi, Maass, & Latrofa, 2017; Shepherd & Evans, 2019). The increase in anger triggered via metadehumanization might thus benefit women in their attempts to change the status quo regarding gender inequalities and to reduce sexual objectification practices. In line with this, previous research on metadehumanization has highlighted its crucial role in the rise of aggressive action tendencies (Andrighetto et al., 2016; Kteily et al., 2016).

Finally, one should notice that online dating is a very specific context. People probably expect to be perceived and evaluated through specific parts of themselves when using dating apps/sites, and particularly through their physical appearance. Similarly, scholars have shown that in some organizational contexts, workers sometimes report organizational metadehumanization because they are valued only for and through their productivity (i.e., Caesens et al., 2017; Caesens et al., 2019). Although being considered only through these specific aspects (i.e., physical appearance and productivity) seems relevant and expected in these two situations (i.e., online dating and organizations), individuals still perceive these evaluations as an aversive experience, lived as a form of interpersonal maltreatment, leading to metadehumanization. In any case, our studies provide strong evidence for the role of sexual objectification in the induction of metadehumanization in interpersonal interactions. Such evidence is consistent with recent literature suggesting that metadehumanization emerges from interpersonal maltreatments. For instance, exploitation, ostracism, betrayal or powerlessness situations all lead to metadehumanization (Bastian & Haslam, 2011; Yang et al., 2015). In organizational settings, links have been found between organizational metadehumanization and abusive supervision (Caesens et al., 2019). Importantly, subtle but frequent forms of interpersonal maltreatments have important consequences for the victims. Indeed, research suggests that exposure to subtle forms of daily discrimination over lifetime (Nadal & Haynes, 2012; Root, 1992) and unwanted sexual advances (Miles-McLean et al., 2015) increase trauma symptoms. Our research sheds light on a mechanism that could explain these effects. Specifically, future research should investigate whether these traumatic consequences might in fact result from the experience of being treated and considered by others as less than a full human being.

### Limitations and Future Directions

We first want to mention that, just as any other meta-perception, metadehumanization may be defined in various ways. Indeed, meta-perceptions’ literature distinguishes intragroup meta-perceptions from intergroup ones. Depending on group membership salience and individual factors (e.g., group identification, awareness of group membership), either intragroup or intergroup meta-perceptions will be formed (Frey & Tropp, 2006). To date, metadehumanization has frequently been defined as the “*perception that one’s own group is perceived by another as less than fully human*” (Kteily et al., 2016, p.3), referring to what could be called metadehumanization at the intergroup level.

With regard to the design we used in the present studies, our participants most probably considered the perpetrator as representing men in general (i.e., the “men” social category) rather than a specific individual. Indeed, we gave almost no other information on the perpetrator except the selected questions and a very short introduction which probably encouraged the depersonalization of the perpetrator (Locksley, Ortiz, & Hepburn, 1980). Similar procedures have been used in other studies in which specific individuals were used as representatives of larger categories. For instance, Kteily and Bruneau (2017) assessed participants’ perceptions that their group is dehumanized by Donald Trump as a representative of the Republican Party. Such perceptions elicited intergroup level metadehumanization responses (see also, Sainz, Martínez, Moya, Rodríguez-Bailón, & Vaes, 2020). In the present studies, however, we cannot exclude that some participants individualized the perpetrator, leading their answers to be related to this person rather than to men in general. Future studies should thus use other methodologies to manipulate female participants’ perceptions that men, in general, objectify them in order to further strengthen the present results. In any case, the interpersonal form of metadehumanization could also be explored in future research as it might likely lead to different consequences than the ones observed at the intergroup level.

Concerning our manipulation of the focus on physical appearance, we used general appearance-related questions asked by an alleged interaction partner, but interpersonal sexual objectification might be expressed through many different forms (e.g., male gaze, appearance-related comments). These different forms of interpersonal sexual objectification could thus induce different types of meta-perceptions and consequences. Indeed, previous studies showed that appearance-related compliments (vs. criticisms) have a key role on self-esteem and body-related consequences (e.g., satisfaction with physical appearance, eating disorders; Calogero et al., 2009; Herbozo & Thompson, 2006). In addition to the specific forms that interpersonal sexual objectification might take, it could also stem from different sources (e.g., romantic partners, family, friends, strangers), and women’s perceptions of appearance-related comments vary according to their interaction partners (Lameiras-Fernández et al., 2018). Future studies are thus needed to explore the varying effects of interpersonal sexual objectification on meta-dehumanization according to the type and source of objectification.

One could question the way we decided to manipulate interpersonal sexual objectification. As noted by Gervais et al. (2020), some studies manipulated interpersonal objectification either through actual or presumed interaction in which women anticipated or believed that they would interact with another (male) partner in a second part of the study. The present studies used another way to focus on people’s physical appearance. Our female participants received sexually objectifying questions from an alleged man who selected questions “*he would like to ask you if you agree to discuss with him*”. We thus believe that our manipulation constitutes a strong way to manipulate sexual objectification in interpersonal interaction. However, other research methods would be necessary to confirm the effect of interpersonal objectification on metadehumanization and negative emotions.

Another important limitation is that we investigated the consequences of interpersonal objectification through a man-woman interaction. As conceptualized by Fredrickson and Roberts (1997), the most classic manifestation of sexual objectification indeed involves a situation in which the focus on physical appearance is delivered by a man to a woman. However, research has shown that women also tend to objectify other women (Strelan & Hargreaves, 2005) and that the frequency and consequences of the exposition to male gaze and sexual harassment do not differ between heterosexual and lesbian women (Hill & Fischer, 2008). Moreover, some studies suggest that men are more and more objectified in media (Hatton & Trautner, 2011; Pope, Olivardia, Gruber, & Borowiecki, 1999), which suggests that they might suffer from similar consequences when exposed to focus on physical appearance behaviors. For instance, Kahalon et al. (2018) found that men experiencing sexual objectification experience a decrease in their cognitive performance. Interestingly, when objectification takes the form of a compliment (rather than the form of questions related one’s physical appearance as is the case in our studies), it leads to an improvement of high TSO participants’ mood, both among males and females. In line with these results, future studies should seek at extending our results to other targets to determine if sexually objectifying practices have the same deleterious consequences on men and women regardless of a perpetrator’s gender or of their own sexual orientation.

Finally, the link observed between metadehumanization and negative emotions is correlational and does not imply causality. Previous studies showed the role of metadehumanization in the occurrence of negative emotions through experimental designs (i.e., Bastian & Haslam, 2011; Zhang et al., 2017), but, as we did not manipulate metadehumanization, we cannot establish the predictive role of metadehumanization in the occurrence of negative emotions. Our mediation analyses should thus be interpreted with cautious and further studies should clarify the relationships between these variables.

## Conclusion

The present paper is the first to explore the consequences of a focus on one’s physical appearance on metadehumanization and negative emotions in interpersonal contexts. Our findings showed that subtle forms of interpersonal objectification increase metadehumanization which in turn elicit anger and sadness. The impact of metadehumanization following interpersonal objectification is still underexplored despite its potential far-reaching consequences. Futures studies should help researchers to better apprehend the behavioral consequences of such negative meta-perceptions on individuals’ psychological functioning and on the way interpersonal interactions evolve.

## Footnotes

In Study 1, we also assessed self-dehumanization and expected to replicate the results highlighted by Loughnan et al. (2017), in which sexually objectified women self-attribute fewer human characteristics. Unfortunately, we did not manage to replicate these effects. The results obtain on self-dehumanization are presented in the online supplement section.

We provide Study 2’s survey flow rather than Study 1’s survey flow. Study 2 used exactly the same manipulation and measurements of Study 1, except for emotions that are not measured in Study 1. This survey flow has the advantage to provide all measurements used across the present studies.

For Study 1, we also assessed “interaction motivation” to meet and discuss with Antoine. For this purpose, we used two items (e.g., “I would be in favor of a meeting with this person if I was in an online dating process”, “I would agree to chat with Antoine if I was in an online dating process”) and participants had to indicate their levels of agreement with each statement on a 7-point Likert scale. One-way ANOVA indicated that participants in the focus on physical appearance condition reported lower motivation levels to interact with and meet Antoine (*M* = 3.30; *SD* = 1.64) compared to participant in the control one (*M* = 4.83; *SD* = 1.68), *F*(1,139) = 29,42, *p* < .001, η^2^_p_ = .17.

In addition, in Studies 2a and 2b, we also thought to explore identity management strategies (Tajfel & Turner, 1979). We expected that interpersonal focus on physical appearance, as a form of gender discrimination, and anger would increase women’s tendencies to engage in collective actions (Chaudoir & Quinn, 2010; Iyer & Ryan, 2009; Leonard, Moons, Mackie, & Smith, 2011; Shepherd & Evans, 2019). As we did not obtain any results on these variables, detailed method and results related to them were moved to the Supplementary Material section.
